# Efficacy and Safety of a Regimen for Medical Abortion up to 63 Days of Gestation: A Retrospective Study in Northern Greece

**DOI:** 10.3390/jcm14041235

**Published:** 2025-02-13

**Authors:** Vera Kelesidou, Ioannis Tsakiridis, Kyriaki Mitta, Andriana Virgiliou, Georgios Michos, Anna Kougioumtsidou, Apostolos Mamopoulos, Themistoklis Dagklis, Ioannis Kalogiannidis

**Affiliations:** Third Department of Obstetrics and Gynaecology, School of Medicine, Faculty of Health Sciences, Aristotle University of Thessaloniki, 54124 Thessaloniki, Greece; verakelesidou@outlook.com (V.K.); kiki93mit@gmail.com (K.M.); giomichos@hotmail.com (G.M.); akougioum@gmail.com (A.K.); amamop@auth.gr (A.M.); dagklis@auth.gr (T.D.); ikalogia@auth.gr (I.K.)

**Keywords:** medical abortion, misoprostol, mifepristone, early pregnancy, first trimester

## Abstract

**Background and Objectives**: First trimester medical abortion with the combination of mifepristone and misoprostol is highly effective. The aim of this study was to assess the efficacy and safety of a modified medical abortion protocol, offering a structured outpatient approach up to 9 weeks (63 days) of gestation. This study may contribute to existing evidence by evaluating a slightly altered regimen and its real-world effectiveness in clinical practice. **Material and Methods**: This was a retrospective cohort study conducted at the Third Department of Obstetrics and Gynecology, School of Medicine, Faculty of Health Sciences, Aristotle University of Thessaloniki, Greece, during a two-year period (2022–2024). Women aged 18 years or older with an intrauterine pregnancy ≤63 days confirmed by transvaginal ultrasound were included. Exclusion criteria included ectopic pregnancy, contraindications to study medications, or loss to follow-up. Mifepristone (200 mg) was administered orally on Day 1, and misoprostol (800 mcg) was administered vaginally on Day 3. Serial follow-up visits on Days 8, 15, and 21 were scheduled according to the success of each cycle. **Results**: In total, 130 women were included in the study. A high success rate of 96.9% (95% CI: 92.3–99.2%) was observed. Success was most frequently observed on Day 8 (60.8%). Success rates were 100% for the age group of 18 to 25 years, approximately 96% for both the groups of 26–35 and 36–45 years, and around 50% for those aged >45 years. With regards to gestational age, success rates were 97.5% for ≤7 weeks (49 days) and 95.9% for >7 weeks (*p* = 0.63). Side effects were reported by 13.2% of participants (95% CI: 7.8–20.1%) and mainly involved the gastrointestinal system. Pain intensity was reported as moderate; analgesics were used by 75.4% of patients. The satisfaction rate for the medical abortion process was 95.4% (95% CI: 90.2–98.3%). **Conclusions**: Early-first-trimester medical abortion on an outpatient basis is a feasible, patient-friendly method with high efficacy, exhibiting limited side effects and a high satisfaction rate.

## 1. Introduction

Worldwide, about 30 million legal abortions are performed per year. Eastern Europe has been documented as having some of the highest abortion rates, while Western Europe reports some of the lowest [1]. In many countries, the availability of accurate data on induced abortions varies, reflecting the diverse legal and cultural contexts in which these procedures take place. Nonetheless, abortion remains a significant public health issue, necessitating ongoing vigilance regarding its safety and psychosocial impact. Greece ranks among the European countries with a relatively high rate of abortions [2], while teenage abortion, in particular, constitutes a significant concern [3]. However, national data on medical abortion remain scarce and access to structured outpatient protocols is inconsistent, highlighting a gap in clinical practice that warrants further investigation. Addressing these issues is crucial for both patient safety and public health.

According to the literature, medical abortion in early pregnancy is as effective as surgical abortion [4]. Beyond its comparable efficacy, medical abortion can be associated with reduced healthcare resource utilization and may offer greater privacy and autonomy for women, thereby enhancing its acceptability in many settings. In particular, medical termination of pregnancy up to 9 weeks (63 days) of gestation using a combination of mifepristone and prostaglandins is recognized as a safe and effective alternative to surgical approaches [5]. Initially, mifepristone alone was used for medical abortion, but its success rate—ranging between 60% and 80%—was considered moderate [6]. This led to the combined administration of mifepristone with prostaglandins, which has since become a standard therapeutic method in many high-resource settings [7,8,9]. Currently, misoprostol, a prostaglandin E1 analogue, is frequently used alongside mifepristone in medical abortion regimens [5]. When administered sequentially, these medications induce cervical ripening and uterine contractions, facilitating a complete abortion with minimal invasive procedures. More specifically, administering mifepristone first and following it 48 h later with misoprostol, has been consistently shown to be both highly effective and well-tolerated for pregnancies of up to 63 days [10,11]. Nevertheless, limited data exist regarding the performance of such a protocol in certain populations and specific healthcare settings, including outpatient clinics. Ongoing research continues to explore optimal dosing intervals and routes of administration to maximize efficacy and patient satisfaction, while minimizing side effects.

Given that Greece faces one of the highest abortion rates in Europe, particularly among adolescents, there is a clear public health imperative to explore safe, patient-friendly, and efficient methods of pregnancy termination. The aim of this study was to evaluate the efficacy and safety of a standardized medical abortion protocol up to 9 weeks of gestation, with a focus on particular outcomes, including side effects and patient satisfaction. By gathering data on the success rates and tolerability of this combined regimen, this study aimed to contribute valuable insights that may inform clinical guidelines and public health policy in Greece and beyond.

## 2. Material and Methods

This retrospective cohort study was conducted at the Third Department of Obstetrics and Gynecology, School of Medicine, Faculty of Health Sciences, Aristotle University of Thessaloniki, Greece, between December 2022 and March 2024.

### 2.1. Study Participants

Women aged 18 years or older seeking medical termination of pregnancy were screened for eligibility. Inclusion criteria included intrauterine pregnancy confirmed by transvaginal ultrasound (TVUS) at ≤63 days of gestation, willingness to return for scheduled follow-up, and no known contraindication to mifepristone or misoprostol. Exclusion criteria included a history of ectopic pregnancy, documented allergies to either medication, and loss to follow-up prior to final outcome assessment.

### 2.2. Ethical Approval and Informed Consent

All participants provided written informed consent after receiving detailed information about the study, including its voluntary nature and guarantees of confidentiality. No incentives were provided. This study was approved (20 February 2024) by the ethics committee of the Aristotle University of Thessaloniki (No.115/2024).

### 2.3. Medical Abortion Protocol

On Day 1 (baseline), each participant underwent clinical and ultrasound evaluation to confirm intrauterine gestation and gestational age, ensuring the presence of a gestational sac within the uterus. Upon meeting the inclusion criteria, 200 mg mifepristone was administered orally, marking the start of the medical abortion protocol. After a 48 h waiting period, participants returned on Day 3. A follow-up ultrasound was performed routinely for all participants to determine if the abortion was incomplete, defined by visualization of a gestational sac or an endometrial thickness > 15 mm. In such cases, 800 mcg misoprostol was administered vaginally. Additional follow-up visits on Days 8 and 15 included repeat ultrasounds, evaluation of side effects (e.g., nausea, vomiting, diarrhea), and careful assessment of bleeding and pain patterns. If ultrasound findings still did not meet the criteria for a complete abortion (absence of gestational sac and endometrial thickness ≤ 15 mm), another 800 mcg dose of misoprostol was administered at each of these visits. By Day 21, if products of conception remained visible or the endometrial thickness surpassed 15 mm, no further medical management was pursued; instead, surgical intervention such as dilation and curettage was planned.

### 2.4. Outcome Measures

The primary outcome was successful completion of abortion without requiring surgery, confirmed by the absence of a gestational sac and an endometrial thickness of 15 mm or less on ultrasound. Secondary outcomes encompassed the monitoring of gastrointestinal side effects, bleeding that exceeded normal expectations, and pain intensity measured on a standardized 0–10 scale; participants were asked to report analgesic usage accordingly. Moreover, patients rated their overall satisfaction with the medical abortion process using a structured 5-point Likert scale (very dissatisfied, dissatisfied, neither dissatisfied nor satisfied, satisfied, very satisfied). These measures, alongside with the scheduled ultrasound assessments and repeat misoprostol doses as necessary, provided a thorough evaluation of both the efficacy and the patient-centered acceptability of this medical abortion regimen.

### 2.5. Data Collection

In order to capture a comprehensive and accurate account of each participant’s experience, all relevant clinical and demographic data—such as age, parity, gestational age, and baseline ultrasound findings—were initially recorded in standardized forms to maintain consistency and reduce the risk of transcription errors. Throughout the study, ultrasound examinations were performed by trained clinicians following a uniform scanning protocol designed to minimize inter-observer variability, thereby enhancing the reliability of imaging results. In addition, each participant was instructed to maintain a daily log documenting bleeding patterns, pain intensity, and any adverse effects, including gastrointestinal symptoms or other complications. This log was meticulously reviewed at each follow-up visit to ensure accurate reporting. By combining data from the standardized forms, the uniform ultrasound evaluations, and the participants’ daily logs and interviews, the research team was able to acquire a detailed and high-quality dataset. All information—ranging from clinical measurements to subjective experiences—was systematically entered into a secure database, accessible exclusively to authorized members of the research team, thereby maintaining the confidentiality of participant information while enabling efficient tracking of study outcomes.

### 2.6. Statistical Analysis

All statistical analyses were performed using R (RStudio interface, version 2023.03.0+386). Descriptive statistics—including means, medians, standard deviations (SDs), and ranges—were calculated for continuous variables (e.g., age, gestational age, bleeding duration, pain intensity). Frequencies and proportions were used to summarize categorical variables (e.g., overall success rates, side effects, satisfaction).

To derive confidence intervals for proportions (such as success rates or side effects), binomial tests were used. Subgroup analyses of categorical variables—such as success by age group, parity, or gestational age—were evaluated primarily with Fisher’s exact test, given that certain cells had small sample sizes. Chi-square tests were applied when expected cell counts were sufficient. A *p*-value < 0.05 was considered statistically significant. No data imputation was performed; participants lost to follow-up were excluded from the final analysis.

Subgroup analyses were performed to explore potential factors influencing the success of the medical abortion protocol. Maternal age was divided into subgroups, parity into nulliparous and multiparous, and gestational age into ≤7 weeks (49 days) and 7–9 weeks (50–63 days). The choice to divide gestational age at 7 weeks was informed by the distribution of cases, which showed a significant concentration around this time point. Fisher’s Exact Test was used to evaluate statistical significance in these subgroups.

## 3. Results

A total of 155 women met the inclusion criteria; however, 25 were lost to follow up; therefore, 130 with complete follow-up were enrolled in the final analysis. The mean age was 30 years (SD = 7.8 years), and the mean gestational age at presentation was 48 days (SD = 6.6 days). The median parity was 2 (range: 0–7), reflecting a diverse pregnancy history among the cohort.

After evaluating the outcomes of the medical abortion protocol, a high success rate of 96.9% (95% CI: 92.3–99.2%) was found. Success was most frequently observed on Day 8 (60.8%), followed by Day 15 (17.7%), Day 3 (13.9%), and Day 21 (4.6%) (Table 1).

Side effects were reported by 13.1% of participants (95% CI: 7.8–20.1%). Apart from the anticipated outcomes of bleeding and pain related to the procedure, these included primarily gastrointestinal symptoms, i.e., nausea, vomiting, and diarrhea. With regard to bleeding and pain, their onset pain typically occurred by Day 3. The mean reported duration of bleeding was 7.3 days (SD = 3.9 days), with a range up to 22 days, while the mean pain duration was 2.8 days (SD = 1.2 days), with a maximum of 8 days observed. Pain intensity had a mean value of 5.6 on a scale of 1 to 10, indicating a moderate level of discomfort. Analgesics were used by 75.4% of patients.

The overall satisfaction rate was high, with 95.4% (95% CI: 90.2–98.3%) of the participants reporting being “very satisfied” or “satisfied”. A smaller percentage of 3% (95% CI: 0.8–7.8%) expressed neutral feelings, while only 1.6% (95% CI: 0.1–6.2%) reported dissatisfaction. These findings highlight the general acceptability and positive reception of the medical abortion protocol used in this study.

A subgroup analysis was performed, detailing the success rates based on various factors (Table 2). The success rates of medical abortion by age group were 100% for the 18–25 years (95% CI: 91.6–100%), 96.4% for 26–35 (95% CI: 87.5–99.6%), 96.8% for 36–45 (95% CI: 83.3–99.9%), and 50% for >45 years (95% CI: 1.3–98.7%); these differences did not reach statistically significant (*p* = 0.08). Of note, only two participants were >45 years.

With regards to parity, nulliparous women achieved a 100% success rate, albeit with a wide confidence interval (95% CI: 66.4–100%) due to the smaller sample size in this subgroup. Multiparous women had a success rate of 96.7% (95% CI: 91.8–99.1%). The analysis did not reveal a statistically significant difference between the parity groups (*p* = 1.0).

Analysis on the effects of gestational age on medical abortion success showed no statistical significance (*p* = 0.63). More specifically, women at a gestational age of ≤7 weeks had a success rate of 97.5% (95% CI: 91.4–99.7%), while those at a gestational age of >7 weeks had a success rate of 95.9% (95% CI: 86.0–99.5%).

## 4. Discussion

The main findings of this study indicate that the combined regimen of mifepristone and misoprostol had a high success rate of approximately 97%. Moreover, most women (60%) achieved abortion between Days 3 and 8, demonstrating the rapid effectiveness of the protocol. Furthermore, neither gestational age nor parity appeared to significantly influence the efficacy, suggesting that the regimen remains reliable across different patient groups. In terms of safety, side effects were reported by approximately one in seven participants, primarily affecting the gastrointestinal system. Despite these minor adverse effects, patient satisfaction with the medical abortion process was notably high, reaching 95%. Overall, these observations underscore the practical advantages of a mifepristone-misoprostol regimen for early pregnancy termination, particularly in an outpatient setting where resource utilization can be optimized.

A recent systematic review, which included 33 studies with a total of 22,275 patients undergoing medical abortion in the early first trimester (≤63 days), highlighted that the combination of mifepristone and misoprostol is associated with fewer ongoing pregnancies, higher success rates, and greater patient satisfaction compared to regimens using only misoprostol [12]. Within these combined regimens, an 800mcg dose of misoprostol was found to be more effective than 400 mcg, while no major differences were reported regarding the timing between mifepristone and misoprostol administration or the method of misoprostol delivery. Serious adverse events occurred infrequently [12,13]. These observations further support our findings, given that our protocol similarly used a 200 mg dose of mifepristone followed by 800 mcg of misoprostol.

In our study, misoprostol was administered 48 h after mifepristone, while data from a randomized controlled trial showed that misoprostol administration on Day 1 may be even more effective than on Day 3 (RR: 1.94; 95% CI: 1.05–3.58) [14]. Most studies limit their follow-up to 14 or 15 days without any intervention; if the initial cycle is unsuccessful, surgical abortion is recommended as the next step [11,15,16]. According to a clinical trial, expulsion of the products of conception occurred within four hours in 93% of the patients that received mifepristone and vaginal misoprostol [11]. Another study supports that 95% of women who received the same medical abortion regimen had a complete abortion by day 7 [17], which is in accordance with the findings of our study; most of the successful outcomes were achieved within the first 8 days post-treatment.

Regarding demographic factors, this study points to a possible decline in protocol efficacy among women over 45 years of age, although only two participants belonged to this category. A study of 377 consecutive medical records similarly found that older age was associated with an increased likelihood of unsuccessful medical abortion [18]. On the other hand, gestational age did not significantly affect success rates in our population, mirroring studies that also found no difference in efficacy based on gestational age for pregnancies up to 63 days when using mifepristone plus misoprostol [18]. The same study suggested that multiparity might undermine effectiveness, whereas our subgroup analysis showed no statistically significant difference between nulliparous and multiparous women. These seemingly conflicting observations highlight the complexity of patient factors, such as hormonal profiles, uterine responsiveness, and prior obstetric history, all of which may affect the outcomes of medical abortion in nuanced ways.

Side effects were reported by 13.1% of the participants and mainly involved the gastrointestinal tract. Pain intensity had a mean value of 5.6 on a scale of 1 to 10, and three out of four patients used painkillers in the present study. Various studies in the literature suggest that gastrointestinal symptoms, such as vomiting, diarrhea, and nausea, are the predominant, which is in agreement with our findings [11,16]. Regarding the pattern of degree of pain in a scale of 0 to 10, according to the literature, most women reported less than 5, after misoprostol use. However, 26% of patients asked for analgesia per os, and 10% received parenteral analgesia [11]. In our study, about 75% of the patients reported receiving painkillers at home. This could be explained by the fact that the abortion was performed on an outpatient basis and the feeling of insecurity may be what led to an increased consumption of analgesics. Despite this higher rate of self-directed analgesic use, the overall pain experiences appear manageable, with no significant adverse events reported. Future research may investigate tailored pain management strategies to further improve patient comfort.

Finally, the satisfaction with the medical abortion process was notably high, with 95.4% of participants reporting being either “satisfied” or “very satisfied” with the procedure. This is in agreement with a previous study, where 93.6% of women reported that they were satisfied or highly satisfied with the medical abortion [16]. As for the outpatient approach, the acceptability has been reported to be as high as 87.4%, and the experience was reported to be either satisfactory or very satisfactory [19]. These high satisfaction levels may stem from the convenience and reduced invasiveness of medical abortion, as well as the avoidance of anesthesia and surgery. High satisfaction rates reinforce the importance of patient-centered care, flexible follow-up schedules, and clear communication regarding possible side effects and pain management options.

To the best of our knowledge, this is the first study to evaluate the success rate, satisfaction rate, and efficacy of early-first-trimester medical abortion in outpatient settings within a Greek population. However, this may introduce bias since the overall sample size remains relatively small, particularly for the subset of older women. It should be noted that although our department is a referral center for a total population of about 1.5 million people, medical abortion on an outpatient basis has only been introduced relatively recently, possibly affecting the uptake and awareness of this service.

## 5. Conclusions

The present study demonstrates that early-first-trimester medical abortion, using mifepristone (200 mg) followed by vaginal misoprostol (800 mcg), is both highly effective and well-tolerated in an outpatient setting. Completion rates approached 97%, with most women experiencing successful expulsion by Day 8, and side effects—predominantly mild gastrointestinal symptoms—were reported by only a minority of participants. In addition to its efficacy, the regimen yielded a high satisfaction rate, suggesting that it is both acceptable and patient-friendly. These findings underscore the growing preference for medical abortion among both patients and providers, especially when adequate counseling and follow-up protocols are in place. The establishment of a safe and efficient medical abortion protocol, especially in early pregnancy, is of great importance, particularly when considering healthcare resource allocation and patient autonomy. Greece is a country with a high abortion rate; therefore, there is an urgent need for efficient and cost-effective protocols to be established. Incorporating standardized pain management guidelines and clear patient education materials could further optimize both clinical outcomes and patient satisfaction. Further large longitudinal studies are needed to confirm our findings and establish such protocols in outpatient settings. Such research should ideally investigate potential variations in success rates among different demographic groups, explore the cost-effectiveness of various medical abortion regimens, and address long-term follow-up to ascertain any delayed complications or adverse outcomes.

## Figures and Tables

**Table 1:** Success rates, side effects, and satisfaction for medical abortion.

Results	Count	Percentage	95% CI
Overall Success Rate	126	96.92	(92.31, 99.16)
Success on Day 3	18	13.85	(81.47, 100)
Success on Day 8	79	60.77	(95.44, 100)
Success on Day 15	23	17.69	(85.18, 100)
Success on Day 21	6	4.62	(54.07, 100)
Side Effects	17	13.08	(7.81, 20.11)
Satisfaction Rate	123	95.35	(90.15, 98.27)

**Table 2:** Success rates of medical abortion by age group, gestational age, and parity.

Group	Success Count	Total Count	Success Rate (%)	95% CI
**Age**				
18–25	42	42	100	(91.6, 100)
26–35	53	55	96.4	(87.5, 99.6)
36–45	30	31	96.8	(83.3, 99.9)
46+	1	2	50	(1.3, 98.7)
**Gestational age**				
>7 weeks	47	49	95.9	(86.0, 99.5)
≤7 weeks	79	81	97.5	(91.4, 99.7)
**Parity**				
Multiparous	117	121	96.7	(91.8, 99.1)
Nulliparous	9	9	100	(66.4, 100)

## Data Availability

Data are available upon request.
